# Severe Polymyalgia Rheumatica-Like Syndrome Induced by Immune Checkpoint Inhibitor: A Case Report and Literature Review

**DOI:** 10.7759/cureus.74474

**Published:** 2024-11-26

**Authors:** Toshiaki Tsurui, Hirotsugu Ariizumi, Yutaro Kubota, Takuya Tsunoda

**Affiliations:** 1 Division of Medical Oncology, Department of Medicine, Showa University School of Medicine, Tokyo, JPN; 2 Division of Medical Pharmacology, Showa University Graduate School of Medicine, Tokyo, JPN

**Keywords:** case report, esophageal cancer, immune checkpoint inhibitor, immune-related adverse events, polymyalgia rheumatica

## Abstract

Immune checkpoint inhibitors (ICIs) have dramatically improved the prognosis of patients with cancers. However, ICIs can provoke systemic toxicities, which are known as immune-related adverse events (irAEs). Polymyalgia rheumatica (PMR)-like syndrome induced by ICI is one of the most common rheumatic irAEs. However, the management of PMR-like syndrome has not been established yet, and the optimal dose of corticosteroids remains unknown, especially for severe cases. A man in his 60s with advanced esophageal squamous cell carcinoma receiving nivolumab and ipilimumab therapy presented with a fever. Subsequently, the patient experienced bilateral femoral pain and progressive fatigue and was unable to stand within a week. The diagnosis of polymyalgia rheumatica (PMR)-like syndrome was made, and high-dose glucocorticoid therapy was started. The symptoms subsided the next day, and the corticosteroids could be quickly tapered into 10 mg over 3 weeks. The patient had sustained disease control 1 year after ICIs were discontinued. High-dose glucocorticoid therapy achieved prompt improvement of symptoms of PMR-like syndrome, even in a severe case, without compromising the anti-tumor effect of ICIs. However, the ideal treatment approach remains unknown, and prospective clinical studies are needed to determine the best strategy, especially in severe cases.

## Introduction

Immune checkpoint inhibitors (ICIs) have significantly improved the survival of patients with cancer and are now widely used across various settings. However, ICIs can provoke systemic immune-related adverse events (irAEs), which deteriorate the patient’s quality of life and can be life-threatening. Rheumatic irAEs are seen in 3-18% of patients and present a variety of symptoms [1,2]. Polymyalgia rheumatica (PMR)-like syndrome induced by ICI is one of the most common rheumatic irAEs. The diagnosis of PMR-like syndrome is challenging for oncologists because the symptoms and laboratory findings may mimic cancer-related symptoms such as fever, general weakness, and weight loss, as well as elevated acute phase reactants, which are commonly seen in patients with advanced cancer. In addition, PMR-like syndrome induced by ICIs may represent different clinical manifestations from *de novo* PMR, and specific diagnostic criteria for PMR-like syndrome are lacking [3,4]. PMR-like syndrome may present more aggressively compared to classical PMR and can be resistant to corticosteroids in some cases [5]. However, the management of PMR-like syndrome has not been established yet, and the optimal dose of corticosteroids remains controversial, especially for severe cases.

Here, we present a case of severe PMR-like syndrome induced by nivolumab and ipilimumab. We successfully treated it with high-dose corticosteroid therapy and could rapidly taper the steroids without compromising the anti-tumor effect.

## Case presentation

A man in his 60s presented with a fever while undergoing chemotherapy for advanced esophageal squamous cell carcinoma with metastasis to the mediastinal lymph nodes and vertebra. He had begun nivolumab and ipilimumab therapy 2 months prior. Initially, he had no associated symptoms, and a physical examination revealed no remarkable findings. Blood tests showed leukocytosis (10,100 white blood cells/μL), an elevated C-reactive protein level (8.7 mg/dL), and a prolonged erythrocyte sedimentation rate (66 mm/h). Two sets of blood cultures were obtained. Oral levofloxacin was prescribed empirically. Subsequently, the patient experienced bilateral femoral pain and progressive fatigue and was unable to stand within a week. These symptoms worsened in the morning and gradually waned in the evening. The patient did not report any headaches or visual changes.

On admission, the patient’s body temperature was 38.3 °C. Other vital signs were normal. At this point, possible differential diagnoses included infections, including deep abscess and endocarditis; rheumatic irAEs, including inflammatory arthritis; PMR-like syndrome; myositis; eosinophilic fasciitis and vasculitis; endocrine irAEs, such as adrenal insufficiency and thyroiditis; and levofloxacin-induced tendinopathy. Physical examination revealed no heart murmurs, restricted joint movements, or tenderness in the peripheral joints, shoulders, hips, or legs. No skin rash was noted, and muscle strength in the extremities was normal. Laboratory tests showed elevated inflammatory marker levels: leukocytes (13,600/μL) and C-reactive protein (27.14 mg/dL). Thyroid and cortisol panels, creatine kinase, rheumatoid factor, anti-cyclic citrullinated peptide antibody, and antineutrophil cytoplasmic antibody were all negative. Computed tomography was notable only for the disappearance of the mediastinal lymph nodes. Magnetic resonance imaging of the thighs and gallium scintigraphy revealed no abnormalities. Repeated blood cultures showed no bacterial growth, and echocardiography revealed no valvular vegetation.

**Figure 1:**
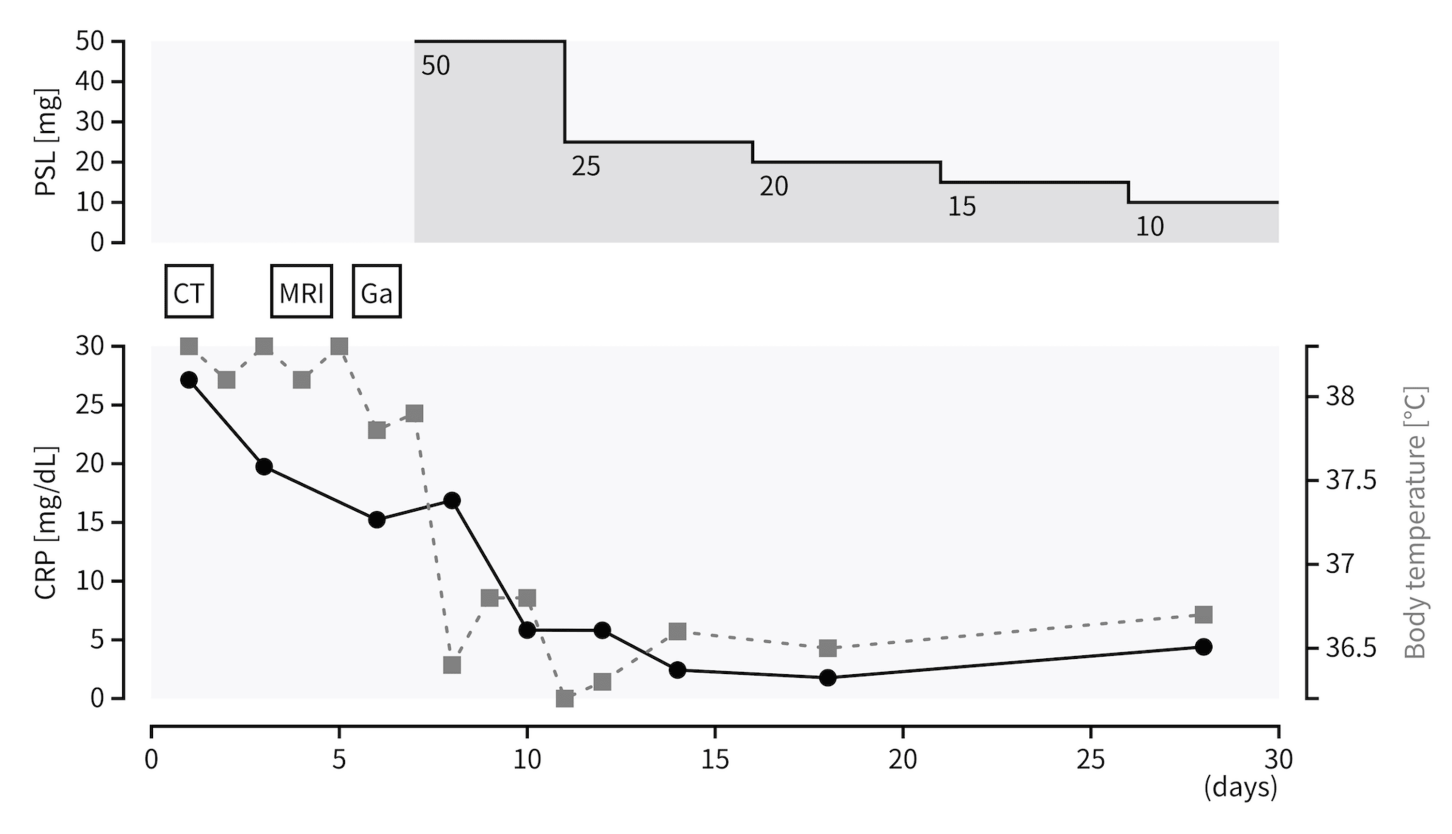
Clinical course after admission. Corticosteroids were administered on day 7, and the fever subsided on the next day. Steroids were rapidly tapered into 10 mg, and the patient was discharged on day 30. In the line chart, the black points signify the CRP level and the gray squares signify the body temperature. CRP, C-reactive protein; CT, computed tomography; Ga, gallium scintigraphy; MRI, magnetic resonance imaging; PSL, prednisolone.

After the withdrawal of ICI therapy and levofloxacin, his fever persisted, and the restriction of daily activity was getting worse. Systemic glucocorticoid therapy with a prednisolone dose of 1 mg/kg was initiated. The patient’s fever subsided the next day, and his fatigue and femoral pain lessened. Although his clinical presentation did not meet the typical features of *de novo* PMR, his clinical course and the rapid response to corticosteroids suggested PMR-like syndrome induced by nivolumab and ipilimumab, especially in the absence of another possible differential diagnosis. The dose of prednisolone could be quickly tapered into 10 mg over 3 weeks until his discharge without any signs of recurrence of the symptoms (Figure 1).

**Figure 2:**
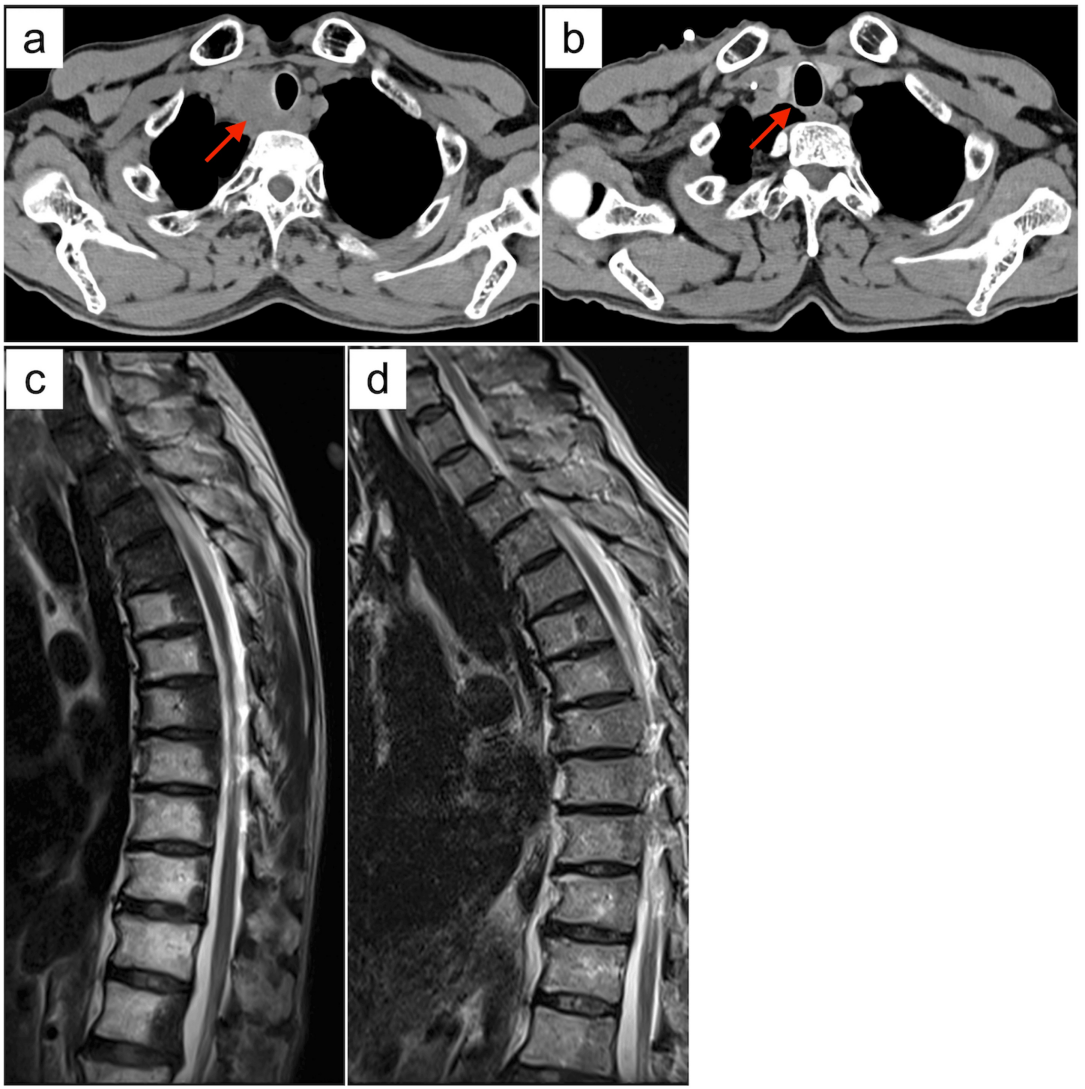
Imaging findings before and after the treatment. Computed tomography shows a mediastinal lymph node metastasis (arrow) directly invading the trachea at baseline (a), which completely disappeared 1 year after ICIs were discontinued (b). Magnetic resonance imaging demonstrates multiple metastases of vertebrae at baseline (c), which were indistinguishable but not totally disappeared 1 year after ICIs were discontinued (d).

Although his symptoms relapsed with morning stiffness in both shoulders, which limited the range of motion after the prednisolone was reduced to 5 mg, these symptoms soon resolved with the increased prednisolone dose. Then, the prednisolone was slowly tapered and withdrawn over 1 year. The patient had sustained disease control 1 year after ICIs were discontinued (Figure 2).

## Discussion

Here, we presented a case of severe PMR-like syndrome induced by nivolumab and ipilimumab. High-dose corticosteroid therapy successfully improved the symptoms, and we could rapidly taper the steroid without compromising the anti-tumor effect.

One of the biggest issues in the management of PMR-like syndrome induced by ICI is represented by the treatment approach. The mainstream treatment consists of systemic corticosteroids; however, the optimal initial dose of steroids remains unclear, especially in severe cases. Major guidelines on the management of irAEs suggest different approaches, which are largely divided into two opposite strategies: “step-up” and “per-grade” approaches (Table 1) [6-9].

**Table 1:** Summary of major guidelines on the management of PMR-like syndrome. ASCO, American Society of Clinical Oncology; csDMARDS, conventional synthetic disease-modifying anti-rheumatic drugs; ESMO, European Society for Medical Oncology; ICI, immune-checkpoint inhibitor; NA, not addressed; NCCN, National Comprehensive Cancer Network; NSAIDs, non-steroidal anti-inflammatory drugs; SITC, Society for Immunotherapy of Cancer; IL, interleukin; TNF-α, tumor necrosis factor-alpha.

	Grade 1	Grade 2	Grade 3	Grade 4
ASCO [6]	Continue ICI. Acetaminophen and/or NSAIDs.	Consider holding ICI. Prednisone 20 mg/day.	Continue ICI. Prednisone 10-20 mg/day. 0.5 mg/kg/day if no improvement.	
ESMO [7]	Continue ICI. Analgesics and/or NSAIDs.	Continue ICI. Prednisone 10-20 mg/day. 0.5 mg/kg/day if no improvement.	Temporarily withold ICI. Prednisone 10-20 mg/day. 0.5 mg/kg/day if no improvement.	Withold ICI. IL-6R inhibitor or TNF-α inhibitor if no response to prednisone 0.5mg/kg/day.
NCCN [8]	Continue ICI. Consider holding ICI if no improvement. Prednisone 10-20 mg/day. Increase to 30–40 mg if no improvement. csDMARDs such as methotrexate, IL-6 inhibitors (tocilizumab or sarilumab) If unable to taper prednisone or no improvement.			
SITC [9]	NA	Prednisone 10-20 mg/day.	Prednisone 40-60 mg/day.	

The European Society for Medical Oncology and the National Comprehensive Cancer Network recommends a "step-up" approach, starting with fixed low-dose corticosteroids (prednisone 10-20 mg) and considering an escalation in dosage or the addition of other immunomodulators, such as methotrexate and interleukin-6 (IL-6) inhibitors, if the response is insufficient. Conversely, the guidelines of the American Society of Clinical Oncology (ASCO) and the Society for Immunotherapy of Cancer (SITC) advocate a "per-grade" approach. This strategy bases the initial steroid dose on the severity grade as defined by the Common Terminology Criteria for Adverse Events (CTCAE). The "per-grade" approach aligns with the management of other common irAEs, such as colitis and hepatitis. To date, no clinical trials have directly compared these two strategies. Therefore, the choice of approach and the initial steroid dose may vary among institutions (Table 2) [10-19].

**Table 2:** Previous cases of PMR-like syndrome with details of treatment. At, atezolizumab; Av, avelumab; D, durvalumab; I, ipilimumab; ICI, immune checkpoint inhibitor; HCQ, hydroxychloroquine, N, nivolumab; NSCLC, non-small cell lung cancer; NE, not evaluable; P, pembrolizumab; T, tremelimumab. The asterisk* signifies that the grade was estimated from the case presentation.

Age	Sex	Types of cancer	Regimen	Grade	ICI	Initial prednisone	Salvage	Reference
77	M	bladder	P	NE	NE	5 mg	no	[10]
73	M	NSCLC	N	NE	conitnued	5 mg	no	[11]
74	M	NSCLC	P	NE	conitnued	7.5 mg	no	[11]
63	F	NSCLC	N	NE	continued	7.5 mg	methotrexate	[11]
59	F	melanoma	N	NE	NE	7.5 mg	no	[5]
69	F	renal cell	N	NE	NE	10 mg	tocilizumab	[5]
81	M	melanoma	P	NE	NE	10 mg	no	[5]
59	M	melanoma	P	NE	NE	10 mg	no	[5]
74	M	renal cell	I + P	NE	NE	10 mg	no	[5]
NE	NE	melanoma	P	G3	NE	10 mg	no	[12]
79	F	NSCLC	N	G3	withdrawn	10 mg	no	[13]
63	F	renal cell	N	NE	withdrawn	10 mg	no	[14]
83	M	urothelial	P	NE	withdrawn	15 mg	no	[11]
72	M	renal cell	N	NE	continued	15 mg	no	[11]
88	M	melanoma	N	G3 *	NE	15 mg	no	[15]
63	F	poorly differentiated	D	NE	NE	15 mg	HCQ	[5]
74	M	melanoma	P	NE	NE	15 mg	no	[5]
65	F	NSCLC	N	NE	NE	15 mg	no	[5]
77	F	melanoma	P	NE	NE	15 mg	no	[5]
NE	NE	melanoma	I + N	G3	NE	15 mg	no	[12]
74	M	melanoma	I	NE	NE	15 mg	no	[1]
74	F	colorectal	P	NE	NE	16 mg	no	[1]
63	M	melanoma	P	NE	withdrawn	20 mg	no	[16]
73	M	prostate	At	NE	NE	20 mg	no	[10]
79	M	melanoma	I + N	NE	NE	20 mg	no	[5]
66	M	melanoma	N	NE	NE	20 mg	methotrexate	[5]
66	M	renal cell	D + T	NE	NE	20 mg	no	[5]
72	F	renal cell	Av	NE	NE	20 mg	no	[5]
66	M	NSCLC	P	NE	NE	20 mg	no	[5]
79	M	melanoma	N	NE	withdrawn	20 mg	no	[17]
75	M	melanoma	N	G3 *	withdrawn	20 mg	no	[18]
69	M	melanoma	I + N	NE	NE	30 mg	no	[5]
68	M	melanoma	I + N	NE	withdrawn	30 mg	no	[17]
65	F	renal cell	N	NE	withdrawn	30 mg	no	[14]
63	M	renal cell	N	NE	NE	40 mg	tocilizumab	[5]
63	M	renal cell	N	NE	withdrawn	40 mg	no	[17]
68	F	renal cell	P	NE	withdrawn	50 mg	methotrexate	[14]
57	M	melanoma	P	NE	NE	60 mg	no	[5]
60	M	melanoma	P	NE	NE	60 mg	no	[5]
88	F	melanoma	P	G3 *	withdrawn	0.3 mg/kg	no	[19]
79	M	melanoma	P	NE	continued	0.3 mg/kg	no	[19]

The "step-up" strategy is a widely accepted method in managing classical PMR and is preferred due to the lower risk of corticosteroid-related adverse effects and its negative impact on anti-tumor efficacy, although the latter remains controversial. This strategy's validity is supported by an observational study where low-dose corticosteroid therapy was found equally effective in PMR-like syndrome [3]. Nevertheless, its effectiveness in severe cases remains uncertain due to the lack of clinical data on severity grades in most reports. Additionally, immune-related adverse events that mimic de novo autoimmune diseases exhibit a varied clinical spectrum, including symptoms, laboratory data, and treatment responses, compared to their non-autoimmune counterparts. The European League Against Rheumatism (EULAR) underscores this aspect in managing rheumatic irAEs [4]. Some instances required adding another immunomodulator to moderate-to-high doses of corticosteroids (average 35 mg) due to resistance [20]. However, these cases alone do not substantiate the "per-grade" strategy, as the link between steroid resistance and disease severity remains unclear.

## Conclusions

In our case, the symptoms progressively worsened and severely restricted the patient’s activity of daily living, which was equivalent to grade 3. We followed the “per-grade” approach, which could rapidly improve the symptoms. We could also quickly taper the corticosteroids and maintain the anti-tumor response at the same time. Prospective clinical studies are needed to determine the best strategy, especially in severe cases.
